# B-Vitamins and Bone Health–A Review of the Current Evidence

**DOI:** 10.3390/nu7053322

**Published:** 2015-05-07

**Authors:** Zhaoli Dai, Woon-Puay Koh

**Affiliations:** 1 Saw Swee Hock School of Public Health, National University of Singapore and National University Health System, Singapore, Block MD1, 12 Science Drive 2, Singapore 117549, Singapore; 2 Office of Clinical Sciences, Duke-NUS Graduate Medical School Singapore, Singapore, 8 College Road Level 4, Singapore 169857, Singapore

**Keywords:** vitamin B, bone, osteoporosis, fracture

## Abstract

Because of ongoing global ageing, there is a rapid worldwide increase in incidence of osteoporotic fractures and the resultant morbidity and mortality associated with these fractures are expected to create a substantial economic burden. Dietary modification is one effective approach for prevention of osteoporosis in the general population. Recently, B vitamins have been investigated for their possible roles in bone health in human studies. In this review, we provide different lines of evidence and potential mechanisms of individual B vitamin in influencing bone structure, bone quality, bone mass and fracture risk from published peer-reviewed articles. These data support a possible protective role of B vitamins, particularly, B2, B6, folate and B12, in bone health. However, results from the clinical trials have not been promising in supporting the efficacy of B vitamin supplementation in fracture reduction. Future research should continue to investigate the underlying mechanistic pathways and consider interventional studies using dietary regimens with vitamin B enriched foods to avoid potential adverse effects of high-dose vitamin B supplementation. In addition, observational and interventional studies conducted in Asia are limited and thus require more attention due to a steep rise of osteoporosis and hip fracture incidence projected in this part of the world.

## 1. Introduction

Nutrient deficiency accelerates bone loss in osteoporosis and increases the propensity to fall, both of which are major causes of hip fracture among elderly [1,2,3]. Although calcium and vitamin D have been most widely studied as the essential nutrients in bone physiology, several reviews have reported that other nutrients may also play important physiological roles in promoting bone health [4,5]. The B vitamins are one group of nutrients that have been investigated for their possible roles in bone health and fracture risk. B vitamins in general are cofactors for the enzymes that are involved in the energy-producing metabolic pathways for carbohydrates, fats and proteins. B vitamins also play an important role in maintaining functions of the nervous system. There are several reviews on B vitamins and bone health with a focus on B9 (folate) and B12 (cobalamin), which are cofactors for the enzymes involved in the remethylation of homocysteine metabolism [6,7,8,9]. However, each specific vitamin B (B1, B2, B3, B6, folate, and B12) has other roles in bone physiology as well.

Inadequate B vitamin intake has been reported among hip fracture patients. The association between various B vitamins (B2, B6, folate, or B12) and a lower risk of osteoporosis or hip fracture has also been demonstrated in several observational studies. The majority of the studies were conducted among Caucasian populations, although the findings are inconsistent among studies or across different B vitamins. In addition, homocysteine has been implicated to increase fracture risk, but whether these B vitamins influence bone health through their links with homocysteine metabolism remains to be determined. However, contrary to the observational findings, most of the clinical trials conducted to date do not appear to support the beneficial effects of B vitamin supplementation in the prevention of osteoporosis or fracture.

In this review, we aimed to provide different lines of evidence from the literature, which include *in vitro* and *in vivo* experimental studies (Table 1), and evidence form observational (Table 2) and intervention studies (Table 3), to summarize the current findings, and attempt to shed light for future research directions in studying the roles of B vitamins in bone health.

**Table 1:** Experimental evidence on B vitamins and bone health.

References	Study Model	Treatment (T) *vs.* Control (C)	Bone Outcomes	Main Findings
Murray *et al.* 1977 [10]	Chick cartilage *in vitro*	T: Isonicotinic acid hydrazide *vs.* C: (G-31H) pyridoxine hydrochloride to chick embryos	Lysyl oxidase activity	Decreased lysyl oxidase activity in cartilage and aorta in treatment chicks *vs.* controls.
Fujii *et al.* 1978 [11]	Rats	T: B6 deficient diet *vs.* C: 30 mg/100 g B6 fed diet	Collagen cross-linking formation	B6-deficient rats had more soluble bone collagens, lower amount of aldehydes and collagen formation.
Bird *et al.* 1982 [12]	*In vitro* embryotic chick aorta	T: PLP added C: no treatment to illuminated lysyl oxidase	Lysyl oxidase activity	The presence of PLP increased enzyme activity. The removal of PLP decreased enzyme activity.
Dodds *et al.* 1986 [13]	Rats	T: B6 deficient diet *vs.* C: 6 mg/kg pyridoxine hydrochloride	Bone formation	Vitamin B6-deficiency reduced G6PD activity in bone formation and callus development. B6-deficient fed rats had more osteoporotic bones with cavities and less new bones.
Masse *et al.* 1994 [14]	Chicks	T: 0.4 mg/kg *vs.* C: 3 mg/kg B6 to young chicks	Bone mechanical property	Deficient chicks had decreased cortical thickness, osteoid in trabecular bone, reduced secondary ossification centers and coarse trabeculation.
Masse *et al.* 1996 [15]	Chicks	T: 0.4 mg/kg *vs.* C: 3 mg/kg of B6	Bone mechanical property and collagen cross-linking	Deficient chicks had decreased fracture load and offset yield load, and increased collagen solubility.
Herrmann *et al.* 2009 [16]	Rats	T: a folate- and B12-deficient diet *vs.* C: standard diet for 12 weeks	Homocysteine level, bone strength (femoral neck compression), bone area, BTM: OC and CTx	Higher plasma homocysteine level in T group *vs.* controls, but no difference for homocysteine concentration in the bone tissue. No difference in bone strength, bone area, or BTM.
Herrmann *et al.* 2007 [17]	Human osteoblasts	Three B vitamins, B6 and folate (µg/L), B12 (ng/L) at various concentrations (0, 0.1, 10, 100, 1000)	Alkaline phosphatase, OC, and P1NP activity; Mineral matrix for formation	No significant difference for BTM levels or mineral matrix among different concentrations of a mix of three B vitamins. Homocysteine level is significantly higher in all concentrations except at the highest concentration of three B vitamins.
Herrmann *et al.* 2007 [18]	Human osteoclasts	Three B vitamins, B6 and folate (μg/L), B12 (ng/L): B6 (0, 2, 4, 6, 35); folate (0, 1, 2.5, 5, 15); B12 (0, 25, 100, 250, 500)	DRA for osteoclast activity, TRAP, and CK activity	Low concentration of the B vitamins combined or alone increased resorption activity significantly *vs.* the highest concentration.
Holstein *et al.* 2010 [19]	Mice	T: folate and B12-deficient diet *vs.* C: control diet for 9 weeks.	Homocysteine, folate, B12, BTM: OC and CTx callus stiffness, size and composition	Folate- and B12-deficient mice showed significantly lower serum folate and B12 but higher serum homocysteine. No significant difference in OC, CTx, callus stiffness and size.

Abbreviations: BTM: Bone turnover biomarker; CK: Cathepsin K; CTx: Carboxyterminal cross-linking telopeptide; DRA: Dentine resorption activity; G6PD: Glucose 6-phosphate dehydrogenase; OC: Osteocalcin; PLP: Pyridoxal phosphate; P1NP: Procollagen type I N propeptide; TRAP: Tartrate-resistant acid phosphatase.

**Table 2:** Observational studies on B vitamins and bone health.

References	Study Populations/Follow-up Time	Exposures	Outcomes	Effect of B Vitamins	Main Findings
**Cross-Sectional Studies**					
Cagnacci *et al.* 2003 [20]	161 Italian postmenopausal women	Serum folate, B12, and homocysteine	BMD	Yes: Folate. No: B12	Folate predicted BMD; No association found in B12 or homocysteine.
Dhonukshe-Rutten *et al.* 2003 [21]	194 free-living Dutch frail elderly people 70 years+	Plasma B12	BMC, BMD, osteoporosis prevalence	Yes: B12 in women	B12 predicted BMC and BMD in women only. Women with marginal or deficient B12 increased risk of osteoporosis substantially.
Golbahar *et al.* 2004 [22]	271 postmenopausal Iranian women	Plasma homocysteine, folate, and B12 ; MTHFR C667T polymorphism	Femoral neck and lumbar spine BMD	Yes: Folate. No: B12	Folate predicted BMD. No correlation between *MTHFR* and folate or B12. Folate but not *MTHFR* predicted plasma homocysteine.
Abrahamsen *et al.* 2005 [23]	1700 Danish postmenopausal women	Dietary intake of B2, B6, folate and B12	BMD	Yes: B2, B6, folate, and B12	B2 is the only significant predictor among the B complex for FN BMD in the TT genotype. Lowest quartile of B2, B6, folate and B12 intake reduced BMD in the TT genotype.
Golbahar *et al.* 2005 [24]	366 postmenopausal Iranian women	RBC 5-MTHFR	BMD	Yes: Folate	RBC 5-MTHF predicted BMD, but not plasma 5-MTHF.
Morris *et al.* 2005 [25]	1500 U.S. White men and women	Serum and RBC folate, serum B12 and homocysteine	BMD; Osteoporosis prevalence risk	Yes: B12; No: Folate	No association was found between folate and BMD or osteoporosis. Significant risk observed in the lowest quartile of B12 *vs.* the highest quartile. A positive relationship between B12 and BMD when B12 < 220 pmol/L.
Baines *et al.* 2007 [26]	328 postmenopausal British women	Plasma homocysteine, serum folate, B6, B12 and MTHFR genotypes	BMD	Yes: Folate; No: B6 and B12	Folate significantly related to BMD. B6, folate and B12 significantly related to homocysteine level. Homocysteine appeared to be related to BMD.
Holstein *et al.* 2009 [27]	94 German men and women	Fasting serum folate, B6, B12	BTM: OC, TRAP; BMD, trabecular thickness, number, and area.	Yes: B6, folate, B12	OC is lower in those with low level of B vitamins. Trabecular thickness and area are lower in those with low folate. Trabecular number is lower in those with low B6. No association between B vitamins and BMD or homocysteine.
Bozkurt *et al.* 2009 [28]	178 postmenopausal Turkish women	Serum homocysteine, folate and B12	BMD at femoral neck and lumbar spine	Yes: B12 ; No: Folate	Homocysteine level was higher in osteoporotic patients *vs.* those with normal or osteopenia. Only B12 predicted osteoporosis at the lumbar spine and femur.
Halıloglu *et al.* 2010 [29]	120 Turkish postmenopausal women	Homocysteine, and serum folate, and B12	BMD, BTM: BAP and CTx	No: Folate and B12	Folate, B12 not related to BMD or BTM. But homocysteine was related to BTM.
Rumbak *et al.* 2011 [30]	131 Croatian women	Plasma homocysteine, serum and red blood cell folate, and B12	BMD from lumbar spine, femoral neck, total femur and distal radius	No: Folate, B12	No relation found for homocysteine, folate or B12 with BMD
**Longitudinal Studies**					
Macdonald *et al.* 2004 [31]	1241 Scottish women aged 45–54 years/6.6 years	Dietary intake of B2, B6, folate and B12	BMD; BMD change; BTM, fPYD/Cr and fDPD/Cr and serum P1NP	Yes: B2; No: B6, folate and B12	B2 intake significantly related to BMD in subjects with *MTHFR TT* homozygotes. No associations found in BMD change or BTM, nor in *CC* or *CT* genotypes. No associations were found in other B vitamins.
Stone *et al.* 2004 [32]	83 U.S. White women 65+ years from a subset/3.5 and 5.9 years	Serum B12	BTM: BAP, osteocalcin; hip BMD and calcaneal bone mass.	Yes: B12	Women at lower serum B12 ≤ 280 pg/mL had higher rate of bone loss from the hip than those at B12 > 280 pg/mL. No association was found with site BMD or BTM.
Dhonukshe-Rutten *et al.* 2005 [33]	615 men and 652 women aged 76 (SD 6.6) years/3 years	Homocysteine, B12 status and the combined effect	Broadband ultrasound attenuation; BTM: OC and of DPD/Cr and fracture risk	Yes: B12	Women with B12 < 200 pM and homocysteine > 15 µM had lower BUA, higher DPD/Cr, and higher OC. No differences in men between the different level of homocysteine and B12. High level of homocysteine and/or low level of B12 increased risk by 2.8 and 3.8 folds in men and women, respectively. Lowest quartile of B12 increased fracture risk in women only. Highest quartile of homocysteine significantly increased fracture risk in men.
Ravaglia *et al.* 2005 [34]	702 Italians aged 65–94 years/4 years	Serum folate, B12 and homocysteine	Fractures	Yes: Folate; No: B12	Higher level of homocysteine increased fracture risk. Lowest folate quartile had 2-fold increased risk *vs.* higher quartiles, but no dose-respondent relationship. No association between B12 and fracture risk.
Tucker *et al.* 2005 [35]	2576 U.S. White men and women 30–87 years	Plasma B12	BMD at total hip, trochanter, Ward’s area, and femoral neck and at lumbar spine (cross-sectional analysis)	Yes: B12	A positive relationship between plasma B12 and BMD at hip in men, and at the lumbar spine for women.
Gjesdal *et al.* 2007 [36]	4766 Norwegian men and women 65–67 years/12.6 years	Plasma homocysteine, folate, B12 and 677C→T and 1298A→C polymorphisms for *MTHFR* genotypes	Hip fracture	Yes: Folate; No: B12	Homocysteine increased hip fracture in both genders. Folate inversely related to hip fracture in women only. No association was found for B12 or *MTHFR* genotypes.
Yazdanpanah *et al.* 2007 [37]	5305 Dutch men and women aged 55+ years/6–7 years	Dietary intake of B2, B6, folate and B12	BMD (cross-sectional analysis); fracture risk	Yes: B2, B6; No: Folate and B12	B2 and B6 positively related to BMD, where B2 is the strongest predictor. Compared to the lowest 3 quartiles of B6, Q4 was related to 23% lower risk in vertebral fracture and 45% lower risk in non- fragility fracture.
Cagnacci *et al.* 2008 [38]	161 healthy postmenopausal women aged 54 years/5 years	Serum folate, homocysteine and vitamin B12.	BMD (cross-sectional and 5 years follow-up)	Yes: Folate No: B12	Initial and 5-year follow-up assessments, as well as annual change of lumbar spine BMD was significantly related to serum folate.
McLean *et al.* 2008 [39]	1002 U.S. White men and women aged 75 years/4 years	Plasma B6, folate and B12, homocysteine	Femoral BMD at baseline and hip fracture	Yes: B6, B12	B6 inversely related to bone loss. B6 and B12 inversely associated with hip fracture risk, and risk remained elevated after adjusted for BMD and homocysteine.
Rejnmark *et al.* 2008 [40]	1869 Danish perimenopausal women 43–58 years/10 years	Dietary intake and supplemented folate, B2, and B12	BMD, fracture risk	Yes: Folate and BMD at 5 years; No: Folate, B2 or B12	No association was found in folate, B2, or B12 with BMD (cross-sectional) or fracture risk. Folate predicted BMD at year 5 significantly.
Yazdanpanah *et al.* 2008 [41]	5305 Dutch men and women aged 55+ years/6–7 years	Dietary intake of B2 and folate	BMD fracture risk	Yes: B2 No: Folate	The lowest quartile of B2 in women with *TT* genotype, bone loss was higher and had 2-fold greater risk in fractures *vs. CC* type. B2 modified *MTHFR C677T* variant on fracture risk. No association was found with folate.
Dai *et al.* 2013 [42]	63154 Chinese men and women 45–74 years/13.8 years	Dietary intake of B1. B2, B3, B6, folate, and B12	Hip fracture risk	Yes: B6; No: other B vitamins	Dose-dependent inverse relationship between B6 and risk of hip fracture in women only. This association was modified by history of diabetes, where the association was present in those without diabetes prevalence.

Abbreviations: BAP: Bone-specific alkaline phosphatase; BMC: Bone mineral content; BMD: Bone mineral density; BTM: Bone turnover biomarker; (f) DPD/Cr: (Free) deoxypyridinoline cross-links/Creatinine; FN: Femoral neck; fPYD/Cr: Free pyridinoline cross-links/Creatinine; MTHFR: Methylenetetrahydrofolate reductase; OC: Osteocalcin; RBC 5-MTHFR: Red blood cell 5-methylenetetrahydrofolate; TRAP: Tartrate-resistant acid phosphatase.

**Table 3:** Randomized clinical trials and meta-analysis on B vitamins and bone health outcomes.

References	Study Populations/Follow-up Time	Treatment (T) *vs*. Control (C)	Outcomes	Effect of B Vitamins on Bone Outcomes	Main Findings
Sato *et al.* 2005 [43]	628 aged 65+ years patients with residual hemiplegia after stroke for 1 year+/2 years	T: Daily treatment with 5 mg folate and 1500 μg B12 for 2 years	Plasma homocysteine, hip fracture incidence	Yes: folate and B12	After two years, treatment group had lower homocysteine and placebo group had higher homocysteine. Hip fracture incidence was significantly reduced in treatment group. No difference in BMD.
Herrmann *et al.* 2006 [44]	61 healthy individuals aged 58 years (SD 8)/8 weeks	T: 0.4, 1 or 5 mg folate daily; C: placebo.	Serum HCY, folate, B12; BTM: OC, P1NP, and CTX.	No: folate	T *vs.* C: Folate increased, homocysteine decreased. No difference in BTM.
Green *et al.* 2007 [45]	267 healthy individuals aged 65 year+/2 years	T: 1 mg folate, 500 μg B12 and 10 mg B6; C: Placebo	Plasma homocysteine, BAP, CTx	No: folate, B6 and B12	Lower homocysteine in T *vs.* C group; no significant difference for serum BAP or CTx
Shahab-Ferdows *et al.* 2012 [46]	132 aged 20–59 years Mexican women/3 months	T: B12 1 mg i.m. then 500/day orally (*n* = 70); C: placebo (*n* = 62).	Serum B12, folate, MMA, holoTC, homocysteine; BTM: BAP	No: B12	Supplementation of B12 increased holoTC and lower MMA and homocysteine. But no difference found in BAP.
Gommans *et al.* 2013 [47]	8164 Caucasian with recent stroke or transient ischemic attack/2.8 year’s therapy and 3.4 years follow-up.	T: Daily 2 mg folate, 25 mg B6 and 500 μg B12 (*n* = 4089); C: Placebo (*n* = 4075)	Fracture risk; Serum homocysteine	No: folate, B6 and B12	No significant difference in fracture risk between two groups. Homocysteine was lower in treatment group, but it did not predict fracture risk.
Keser *et al.* 2013 [48]	31 Croatian women aged 65 years+ with homocysteine >> 10 μmol/L/4 months	T: 800 μg folate and 1000 μg B12 (*n* = 17) daily, C: Placebo (*n* = 14)	BTM: ALP and CTx	No: folate and B12	Treatment group had significantly lower homocysteine and higher serum folate or B12; But no difference in serum level of ALP or CTx.
van Wijngaarden *et al.* 2014 [49]	2919 Dutch aged 65 years with elevated homocysteine (12–50 mmol/L)/2 years	T: Daily 500 µg B12 and 400 µg folate with 600 IU D3 C: placebo with 600 IU D3.	Serum homocysteine, First time fracture	Yes: folate and B12 in 80 years+	Homocysteine significantly lower in the treatment group. Fracture risk did not differ between two groups. But significant lower risk was found in those 80 years+ in the treatment group. However, a higher risk of cancer also observed in the treatment group.
**Meta-Analysis**					
van Wijngaarden *et al.* 2013 [50]	14 cross-sectional studies, and 13 prospective cohort studies and 1 RCT	B12, folate, homocysteine	BMD, fracture risk	Yes: B12, homocysteine	4% borderline significant decreased fracture risk per 50 pmol/L increase in B12, which was (RR: 0.96, 95% CI: 0.92, 1.00); 4% increased risk per mol/L increase in homocysteine (RR: 1.04, 95% CI: 1.02, 1.07). No association between folate and fracture risk. No association was found between folate/B12/homocysteine and BMD in women.

Abbreviations: ALP: alkaline phosphatase; BAP: Bone-specific alkaline phosphatase; BMD: Bone mineral density; BTM: Bone turnover biomarker; CTx: Carboxyterminal cross-linking telopeptide; HCY: Homocysteine; holoTC: Holotranscobalamin; IU: International Unit; MMA: Methylmalonic acid; OC: Osteocalcin; P1NP: Procollagen type I N propeptide.

## 2. Vitamin B1 (Thiamin)

Vitamin B1, or thiamin, in the active form, thiamin pyrophosphate (TPP), is an important cofactor for the key enzymes involved in the metabolism of carbohydrates, lipids and amino acids, and in the synthesis of neurotransmitters [51]. In older individuals, B1 deficiency can become a problem due to reduced appetite and difficulty in eating [52]. The Recommended Dietary Allowance (RDA) for the United States (U.S.) is 1.2 mg for adult males and 1.1 mg for adult females [53]. Good food sources of B1 include whole-grain foods, wheat germ and yeast extract, and pork meat products [52]. 

The evidence on thiamin and bone health is scanty. One study among orthopedic patients reported that thiamin status was deficient among patients with femoral neck fracture but not among those who were admitted for elective total hip replacement [54]. In the Singapore Chinese Health Study, dietary intake of thiamin was not associated with risk of hip fracture in either men or women [42]. A recent review emphasized the major role of thiamin in metabolic function in cells and the consequent impairment of neuro-function in thiamin deficiency. Because thiamin deficiency can impair energy metabolism due to mitochondrial dysfunction in focal regions of the brain [55], this in turn can increase the risk of Alzheimer’s disease and cardiac failure, and therefore can increase the propensity to fall in the elderly [55,56], which may lead to increased fracture risk.

## 3. Vitamin B2 (Riboflavin)

The most important biologically active forms of vitamin B2 or riboflavin are flavin adenine dinucleotide (FAD) and flavin mononucleotide (FMN), both of which participate in the oxidation-reduction reactions in the metabolic pathways that are involved in energy production [57]. Due to aging and reduced efficiency in absorption, elderly individuals are prone to have biological deficiency of B2 [58,59]. The U.S. RDA is 1.3 mg for adult males and 1.1 mg for adult females [53]. The food sources that contribute to riboflavin include cereals, meat, fatty fish, and dark-green vegetables. In addition, milk and dairy products fortified with B2 are the biggest dietary contributors in Western populations [60].

### 3.1. Evidence from Experimental Studies

An early study that examined the offspring of rats fed with B2-deficient diets reported that maternal B2 deficiency induced abnormal fetal development and skeletal malformation [61]. Another *in vitro* experimental study showed that B2 and its photoproducts exerted beneficial effects on cell proliferation and alkaline phosphatase activity, and decreased the RANKL (receptor activator nuclear factor-κB ligand)/OPG (osteoprotegerin) ratio by enhancing the expression of OPG in the preosteoblastic MC3T3-E1 cells [62]. These experimental studies indicate the important roles of B2 as a coenzyme in major metabolic pathways for energy production and its biological properties in bone cells.

### 3.2. Evidence from Observational Studies

Not many studies have examined the associations between B2 and bone health outcomes. Three studies conducted among Caucasian populations looked at dietary intake of B vitamins and found an interaction between the methylenetetrahydrofolate reductase *(MTHFR) C677T* gene polymorphism and B2 intake in relation to bone mineral density (BMD) or fracture risk [23,31,41]. Although dietary intake of B vitamins [23,31,41], BMD [31,41] or fracture risk [41] did not differ across the three genotypes (*CC*, *CT* and *TT*) of the *MTHFR* gene polymorphism, there was an interaction between the *MTHFR* gene polymorphism and B2 associated with BMD [23,31] and fracture risk in women [41]. Among the Scottish and the Danish women, BMD of the femoral neck and lumbar spine were the lowest in women with the lowest quartile intake of riboflavine and *TT* genotype [23,31]. In the Rotterdam study, the highest risk of fracture was also reported among women who were in both the lowest quartile intake of B2 and the *TT* genotype [41]. The proposed mechanism on riboflavin and bone health is related to the coenzymatic form flavin adenine dinucleotide (FAD), which is a cofactor for the *MTHFR* enzyme. Studies suggest that the reduced activity of the so-called thermolabile *MTHFR* enzyme seen in the *TT* genotype is due to the inappropriate loss of its B2 cofactor, and that individuals with the *TT* genotype in turn have reduced activity of the *MTHFR* enzyme only in the presence of insufficient B2 status [63]. Since the highest level of homocysteine was found in these participants with both the *TT* genotype and poor B2 intake [41,63], B2 may interact with the *MTHFR* gene polymorphism to affect bone cells via the effects of hyperhomocystinemia [64].

## 4. Vitamin B3 (Niacin)

Vitamin B3 or niacin is the generic form for nicotinic acid and nicotinamide, both of which are substrates for the active coenzymes, nicotinamide adenine dinucleotide (NAD) and NAD phosphate (NADP). Both coenzymes are important electron acceptors or hydrogen donors for fuel molecules in the redox reactions. NAD also provides substrates for the biological processes involved in DNA processing, cell differentiation, and cellular calcium mobilization [65]. The biosynthesis of B3 in humans is through the tryptophan-niacin conversion, where its efficiency depends on other nutritional and hormonal factors, such as vitamin B6, B2, and iron, all of which are cofactors for the enzymes in the conversion pathway. Nevertheless, humans also obtain B3 from diet. Rich food sources include yeast, meats, cereals, legumes, and seed. An appreciable amount can be found in milk, green leafy vegetables, fish, coffee and tea [65]. The U.S. RDA is 16 mg for adult males and 14 mg for adult females [53].

### 4.1. Evidence from Experimental Studies

Very few studies have examined the direct effect of B3 on bones. Two early studies from the same lab supplemented B3 at different doses to the basal diet to study the effect of B3 on bone strength and mineral content of the tibia in young (one-day-old) [66] and old (six-month-old) chicks [67]. Both studies did not find significant difference in the chick bone mineral content. However, a higher dosage of B3 weakened bone strength and increased bone breakage in both age groups of chicks [29,30], suggesting that excessive niacin consumption may increase bone fractures, although the mechanism has not been clearly elucidated.

### 4.2. Evidence from Observational Studies

A cross-sectional study among Japanese women showed a positive significant correlation between dietary intake of B3 and BMD in premenopausal women [68]. Another case-control study among postmenopausal Korean women suggested that the lowest risk of osteoporosis was found in women in quartile two *versus* the lowest quartile intake of niacin, but there was no significant difference in risk among women in the two upper quartiles [69]. However, due to the small number of participants and inherent biases from the study design, such as selection bias, recall bias, and reverse causality, these results need to be validated by prospective studies. In the Singapore Chinese Health Study, no apparent association was found between B3 intake and risk of hip fracture. In fact, men at quartile three intakes had significantly increased risk of hip fracture compared to those in the lowest quartile [42]. Altogether, these data suggest that supplementation of niacin among elderly deserves further investigation due to the possible adverse effect on fracture risk.

## 5. Vitamin B6 (Pyridoxine)

Vitamin B6 includes three pyridines: pyridoxine, pyridoxal and pyridoxamine, and their 5’-phosphorylated derivatives. The bioactive form is pyridoxal phosphate (PLP), which is involved as a cofactor in over 100 enzymatic reactions in the metabolism of glycogen, phospholipids, and amino acids. PLP plays important roles in neurotransmitters, in the metabolism of amino acids, homocysteine and cystathione, and in the one-carbon metabolism to affect nucleic acid biosynthesis and functioning of the immune system [70]. Although deficiency of B6 is uncommon, elderly individuals and alcoholics are at risk due to reduced appetite and malabsorption. The U.S. RDA for adults over 50 years of age is 1.7 mg/day for men and 1.5 mg/day for women [53]. Good dietary sources include meats, whole grain products, vegetables, bananas, and nuts.

### 5.1. Evidence from Experimental Studies

Earlier work has been conducted in animals to examine the independent role of B6 in lysyl oxidase activity, collagen cross-linking formation, and bone mechanical property Murray, 1977 #295; Fujii, 1979 #63; Bird, 1982 #155; Masse, 1994 #168; Masse, 1996 #86}. As a cofactor for lysyl oxidase, B6, more specifically PLP, is essential for the enzymatic action of lysyl oxidase in collagen cross-linking formation [11,15]. In chick cartilage [10] and chick aorta [12] *in vitro* models, deficiency of B6 reduced the activity of lysyl oxidase, which is an enzyme that catalyzes the oxidative deamination of lysine residues in the tropoelastin and tropocollagen to form aldehydes for the crosslinks in the collagen [12]. This may explain why B6 deficiency could affect the mechanical property of the bone. For example, B6-deficient fed chicks were reported to have decreased fracture load and offset yield load [15], and reduced cortical thickness, trabecular osteoid and coarse trabeculation [14]. Besides its role in lysyl oxidase, other researchers proposed that B6 may act as a substrate of alkaline phosphatase in bone formation [71], and play a role in the coupling between osteoblasts and osteoclasts, where calcification depends on periosteal glucose 6-phosphate dehydrogenase (G6PD) activity, which is induced by putrescine, a compound derived from a B6-dependent enzyme, ornithine decarboxylase [13]. B6 deficiency may thus cause changes in the bone, suggesting an imbalance in the coupling between osteoblasts and osteoclasts with increased bone cavities and reduced new bone formation [13].

### 5.2. Evidence from Observational Studies

Epidemiologic studies relating B6 to bone health or fracture risk are limited. An early report stated that hip fracture patients had significantly lower serum PLP compared to the fit ambulant outpatients [72]. Three prospective cohort studies thus far have found independent roles of B6 in bone mass and/or fracture risk [37,39,42]. The Rotterdam Study showed that lower dietary intake of B6 was associated with lower BMD and higher fracture risk [37], while the Framingham Osteoporosis Study reported that lower plasma B6 level was associated with higher bone loss and hip fracture risk [39]. The role of vitamin B6 in fracture risk appeared to be independent of BMD and homocysteine, because further adjustment for these two variables did not affect the risk estimates substantially [37,39]. In the Singapore Chinese Health Study, dietary intake of B6 was inversely related to risk of hip fracture after adjusting for other risk factors [42]. A cross-sectional study conducted by Holstein and colleagues [27] on a small sample of patients undergoing hip arthroplasty reported that lower serum levels of folate, vitamin B6 and B12 were significantly associated with lower serum level of osteocalcin (bone formation marker). Decreased vitamin B6 and folate levels were associated with weakened bone structure indicated by reduced trabecular number and thickness. However, no associations were found between B vitamins and BMD or homocysteine. They concluded that deficiency in B6 and folate may be related to altered bone properties independent of homocysteine [27].

In the Singapore Chinese Health Study, we found a dose-dependent inverse association between dietary intake of B6 and hip fracture risk among women but not among men. Furthermore, this association was present among women without a history of diabetes, but was attenuated and non-significant among women with diabetes. This finding indicated that gender and diabetes might modify the influence of B6 in risk of hip fracture [42]. B6 may act as a regulator of the steroid hormones, including estrogen, through the modulation of the transcriptional activation to regulate the physiological actions of the hormone receptors [73,74]. As estrogen plays a substantial role in bone turnover [75] and B6 may potentially modulate estrogen, this could explain, at least in part, the protective effect of B6 on hip fracture risk was observed only in women and not in men. Diabetes mellitus is a risk factor for hip fracture by altering the biomechanical integrity of bone and leading to mechanical deterioration and decrease in bone strength [76,77]. We hypothesized that the protective mechanisms by B6 may not compensate for the aforementioned factors detrimental to bone health in patients with diabetes, and this hypothesis needs to be validated in further studies.

## 6. Vitamin B9 (Folate)

Vitamin B9, better known as folate, plays a central role in the one-carbon metabolism in the nucleotide synthesis, in the metabolism of homocysteine, as well as in the methylation of DNA, RNA, proteins and phospholipids [78,79]. As several B vitamins are involved in the metabolism of homocysteine, we are providing more detail about this pathway here. In the methylation pathway, the synthesis of methionine depends on both folate and B12 for remethylation. Thus, deficiency of either B vitamin would result in megaloblastic changes in the bone marrow and other tissues. Alternatively, homocysteine may undergo the transsulfuration pathway to generate cysteine, which depends on B6 as a cofactor for the enzymatic reaction from cystathionine to cysteine [78]. Deficiency of folate in elderly is rare; the U.S. RDA for folate is 400 μg/day for both adult men and women, and a higher dosage of 600 μg/day is required for pregnant women [53]. Major food sources of folate include citrus fruits, dark green leafy vegetables, and legumes [78].

### 6.1. Evidence from Experimental Studies

Several *in vitro* and *in vivo* studies from the same group of researchers have examined the combined effects of deficiency in B vitamins (B6, folate and B12) on the activity of osteoblasts and osteoclasts, bone turnover biomarkers, bone strength and area, and fracture healing [16,17,18,19]. These studies were based on the hypothesis that deficiency of one or more B vitamins might drive higher level of homocysteine, which, in turn, has adverse effects on bone health. The results were generally consistent and showed that although B vitamin deficiency generated significantly higher serum level of homocysteine, there was no significant effect on bone strength and bone area [16], mineral matrix [17], callus stiffness, size or tissue composition [19] or bone turnover [16,17,19]. Since that hyperhomocysteinemia may induce a tissue-specific accumulation of homocysteine in bone through its binding to collagen in the extracellular matrix could adversely reduce bone formation and bone strength [80], these studies suggest that B vitamin deficiency may affect the anatomical/biochemical properties of bone if it is profound enough to cause hyperhomocysteinemia that is sufficient to induce an accumulation of homocysteine in the bone tissue.

We have only found one study thus far that investigated the effect of folate alone in osteoclast cultures [18]. In the folate-deficient treated osteoclasts, resorption activity was found to be significantly increased as compared to the folate treated cells [18]. Furthermore, low folate, B12 and B6 concentrations in osteoclasts resulted in accumulation of homocysteine and stimulation of resorption activity of osteoclasts *in vitro*, including increased dentine resorption activity, tartrate-resistant acid phosphatase, and cathepsin K activity [18], but did not influence the activity of human osteoblasts *in vitro* [17]. Alternatively, folate was suggested to have a comparable effect as tetrahydrobiopterin, a cofactor for the enzyme of nitric oxide synthase; the endothelial isoform of nitric oxide synthase in turn promotes the maintenance of bone density and is a key mediator of the anabolic effects of mechanical loading and estrogens on the bone. Folate may, thus, aid maintenance of bone density by helping to preserve optimal nitric oxide synthase activity in the bone cells [81].

### 6.2. Evidence from Observational Studies

The effect of folate on BMD or fracture risk is not consistent across studies from different populations. Interestingly, one study among Iranian women suggested that red blood cells (RBC) 5-methyltetrahydrofolate (MTHF), which reflected a long-term status of folate in the body, may be a stronger predictor for BMD as compared to short-term markers, such as plasma folate or 5-MTHF [24]. Several epidemiologic studies have found a significant relationship between increased folate intake/level and increased BMD [20,22,24,26,38,40] or reduced fracture risk [34,36]; while others, including our study in the Singapore Chinese Health Study cohort, have shown null associations [25,29,42]. Among these studies, two prospective studies examined dietary intake of B vitamins on BMD [40] and fracture risk [40,42]. Although the study among perimenopausal women from the Danish Osteoporosis Prevention Study showed that dietary folate was a significant predictor for BMD measured at five years after the baseline [40], neither this study nor our findings from the Singapore Chinese Health Study showed that folate from the diet was related to fracture risk [40,42].

## 7. Vitamin B12 (Cobalamin)

The discovery of vitamin B12 or cobalamin started with the supplementation of this vitamin as the unique treatment of pernicious anemia and demyelinating lesions of the central nervous system in 1855 [82]. In fact, B12 was first revealed to be related to osteoporosis and fractures in patients with pernicious anemia [83,84]. Physiologically, B12 is linked to the action of two enzymes, l-methylmalonyl-coenzyme A (CoA) mutase and methiononine synthase [82], the latter enzyme is involved in the metabolism of homocysteine. In elderly individuals, mild to moderate deficiency of B12 can be found in 20% of the population due to malabsorption because of gastrointestinal problems [82]. Elderly deficient in B12 may also present with impaired cognition [85]. The U.S. RDA is 2.4 μg per day for adults 50 years or above [53]. The major food sources include meats, dairy products, eggs, and food products enriched with B12 [82].

### 7.1. Evidence from Experimental Studies

The mechanistic pathways between B12 and different aspects of bone physiology are still unclear despite the evidence from *in vitro* or *in vivo* models. One study using human bone marrow stromal osteoprogenitor cells and UMR 106 osteoblastic cells suggested that B12 increased osteoblastic proliferation and alkaline phosphatase activity, implying a direct effect of B12 on proliferation and formation in the osteoblasts [86]. By contrast, another *in vitro* study showed no direct effects of B12 on osteoblasts (human mesenchymal stem cells) or osteoclasts from the mouse bone marrow regarding cellular differentiation, formation or resorption activity, and matrix calcification [87]. However, B12 deficiency increased the level of homocysteine and methylmalonic acid (MMA), which in turn, stimulated osteoclastogenesis *in vitro* [87]. The authors suspected that B12 deficiency may thus indirectly increase osteoclast formation through its effect on the elevation of MMA and homocysteine levels. As mentioned in the previous section, several investigators have used a combination of B12 with folate and B6 to examine the effect of B vitamins on osteoblasts and osteoclasts *in vitro* and on bone quality in animal models [16,17,18], and showed that deficiency of these vitamins, including B12, increased resorption activity of osteoclasts [18].

### 7.2. Evidence from Observational Studies

The associations between B12 alone or with other B vitamins and bone health outcomes have been studied extensively in different populations, although the majority was conducted among Caucasian populations. Among the 19 studies we reviewed, 12 did not find a significant association between B12 and BMD [20,22,26,29,30,31,37,38], bone turnover biomarkers [29,31] or fracture risk [34,36,37,40,42], regardless whether B12 was measured in blood samples [20,22,26,29,30,34,36,38] or from the assessment of dietary intake [31,37,40,42]. The remaining seven studies [21,25,28,32,33,35,39], however, supported the protective role of B12 in preserving BMD [21,25,28,32,35] and reducing fracture risk [33,39]. Based on the mixed results from these studies, the mechanism of B12 in bone health remains to be elucidated. It has been suggested the BMD only partially explained the relationship between B12 and fracture risk in epidemiologic studies [39]. For example, deficiency of B12 deficiency could contribute to neurological complications [82] to increase the risk of falls in the elderly. Other plausible explanations include the effect of B12 on BMD via its influence on homocysteine metabolism [25,33], but this may not be sufficient to affect bone quality if there is no accumulation of homocysteine in the bone tissues [16].

## 8. Evidence on B Vitamins from Intervention Studies and Meta-Analyses

Despite conflicting results from observational studies, a few randomized clinical trials have been conducted to examine the effects of B6, folate and B12 supplementation in association with changes in bone turnover biomarkers [44,45,46,48] and fracture risk [43,47,49]. To keep in mind, dosage used in these studies ranged considerably among different trials. Although all of the trials showed a successful reduction of homocysteine level in the B vitamin (single or mix B vitamin(s)) supplementation treatment group, none of these studies showed significant changes in levels of bone turnover biomarkers or fracture risk. Only one study reported that combined treatment with folate and B12 significantly reduced risk of hip fracture in Japanese patients following a stroke [43]. Of note, in this Japanese study, no significant difference was found in BMD between the treatment and the placebo groups and bone turnover markers in the blood were not measured for comparison [43]. It appears that the beneficial effect of folate and B12 in the Japanese patients with residual hemiplegia following stroke could be through the improvement in the neurological and cognitive function among these patients, who were also at high risk to have severe neurological deficits and increased the propensity to fall. Another study conducted among a large cohort of Dutch elderly with elevated homocysteine showed that combined B12 and folate supplementation had no effect on osteoporotic fracture incidence in this elderly population. Furthermore, although exploratory subgroup analyses suggested a beneficial effect on osteoporotic fracture prevention in the elderly aged 80 years or above, the supplementation of B12 and folate was associated with increased incidence of cancer [49].

A recent meta-analysis systematically evaluated the effect of folate, B12 or homocysteine on fracture risk and BMD among 27 cross-sectional and longitudinal studies. The authors did not find a statistically significant relationship with BMD, but reported a modest effect of B12 and homocysteine levels on fracture risk. The pooled analysis showed a 4% borderline significant decrease in fracture risk per 50 pmol/L increase in B12 concentration and a 4% statistically significant increase in risk of fracture per 1 mol/L increase in homocysteine level [50]. Together, these findings from the clinical trials and meta-analyses imply a possible but modest effectiveness of B vitamins in bone protection. An optimal dosage for the efficacy of B vitamins in bone outcomes remains to be determined in human studies.

## 9. Other B Vitamins

Choline is an essential vitamin and is usually grouped with the vitamin B complex due to the interconnection between choline and the other B vitamins, such as folate, B6 and B12, in the transmethylation metabolic pathways as methyl donors [88]. The *de novo* biosynthesis of choline is through the methylation of phosphatidylethanolamine to phosphatidylcholine, although *de novo* synthesis alone may not meet the human requirements. Choline can also be acquired from dietary sources, such as milk, liver, eggs, and peanuts [53]. The adequate intake for adults aged 19 years and above is 425 mg per day for women and 550 mg per day for men [53].

The primary function of choline is being a precursor for acetylcholine, phospholipids and betaine [53]. However, evidence examining the relationship between choline and bone outcomes is scanty. One study showed that rats fed with methionine–choline deficient diet had lower trabecular and cortical bone mass due to decreased bone formation and increased bone resorption [89]. A recent and the only observational study so far reported that plasma choline at the lowest tertile was significantly related to low femoral neck BMD in both men and women; while in elderly women aged 71 to 74 years, low plasma choline was significantly associated with an increase in risk of hip fracture, where the highest risk was found among elderly female smokers [90]. Further studies are required to evaluate the effect of choline on bone health.

## 10. Conclusions

Current evidence from the experimental studies provides several mechanistic pathways for the effects of B6, folate and B12 on bone physiology. The results from observational studies are inconsistent in the associations between these B vitamins and bone outcomes, however, majority of the randomized clinical trials have not shown protective effects of B6, folate or B12 in bone turnover or fracture risk reduction. Future clinical studies may include intervening with dietary regimens that are enriched with B vitamins to examine their effects on bone mineral density, bone turnover and/or fracture risk in elderly individuals, and, thus, avoid the potential adverse events from B vitamin supplementations. In addition, observational and interventional studies conducted among Asian populations are scarce and thus require more attention, particularly due to the expectant increase of hip fracture incidence in Asia in the near future.
